# Predicting cardiovascular health trajectories in time-series electronic health records with LSTM models

**DOI:** 10.1186/s12911-020-01345-1

**Published:** 2021-01-06

**Authors:** Aixia Guo, Rahmatollah Beheshti, Yosef M. Khan, James R. Langabeer, Randi E. Foraker

**Affiliations:** 1 Institute for Informatics (I2), Washington University School of Medicine, 600 S. Taylor Avenue, Suite 102, St. Louis, MO 63110 USA; 2 Department of Computer & Information Sciences, Data Science Institute, University of Delaware, Newark, DE USA; 3 Health Informatics and Analytics, Centers for Health Metrics and Evaluation, American Heart Association, Dallas, TX USA; 4 School of Biomedical Informatics, Health Science Center at Houston, The University of Texas, Houston, TX USA; 5 Department of Internal Medicine, Washington University School of Medicine, St. Louis, MO USA

**Keywords:** Cardiovascular health (CVH), The guideline advantage (TGA), LSTM models, CVH prediction, Precision medicine

## Abstract

**Background:**

Cardiovascular disease (CVD) is the leading cause of death in the United States (US). Better cardiovascular health (CVH) is associated with CVD prevention. Predicting future CVH levels may help providers better manage patients’ CVH. We hypothesized that CVH measures can be predicted based on previous measurements from longitudinal electronic health record (EHR) data.

**Methods:**

The Guideline Advantage (TGA) dataset was used and contained EHR data from 70 outpatient clinics across the United States (US). We studied predictions of 5 CVH submetrics: smoking status (SMK), body mass index (BMI), blood pressure (BP), hemoglobin A1c (A1C), and low-density lipoprotein (LDL). We applied embedding techniques and long short-term memory (LSTM) networks – to predict future CVH category levels from all the previous CVH measurements of 216,445 unique patients for each CVH submetric.

**Results:**

The LSTM model performance was evaluated by the area under the receiver operator curve (AUROC): the micro-average AUROC was 0.99 for SMK prediction; 0.97 for BMI; 0.84 for BP; 0.91 for A1C; and 0.93 for LDL prediction. Model performance was not improved by using all 5 submetric measures compared with using single submetric measures.

**Conclusions:**

We suggest that future CVH levels can be predicted using previous CVH measurements for each submetric, which has implications for population cardiovascular health management. Predicting patients’ future CVH levels might directly increase patient CVH health and thus quality of life, while also indirectly decreasing the burden and cost for clinical health system caused by CVD and cancers.

## Background

Cardiovascular disease (CVD) is the leading cause of mortality for both men and women in the United States (US), and accounts for almost 1 in every 4 deaths (https://www.medicalnewstoday.com/articles/282929.php). Cardiovascular health (CVH) metrics, defined by the American Heart Association (AHA), have important implications for CVD prevention [1–6]. Individuals with better CVH metrics have lower risk of CVD death and prevention efforts should focus on maintaining or improving CVH across the lifespan (https://www.medicalnewstoday.com/articles/324195.php). Management of CVH levels according to previously recorded measurements may be critical to better manage CVH level of patients, as closer attention to certain risk factors may maximize prevention. Thus, predicting future CVH levels may be associated with better management of CVD.

The seven cardiovascular risk factors which comprise CVH include: smoking status (SMK), physical activity, body mass index (BMI), diet, blood glucose, cholesterol, and blood pressure (BP) [2]. Recent studies [7, 8] have used previous measurements of weight and height data to predict BMI by employing regression analyses on population survey data. Other recent studies predicting changes in BP, [9, 10] hemoglobin A1c (A1C) [11], cholesterol (LDL) [12], and SMK [13] utilized electronic health record (EHR) data. These studies employed machine learning algorithms such as classification tree, feature selection algorithms, and correlation analyses.

Recently, healthcare organizations have employed deep learning models to discover useful patterns from the EHR, which contain rich longitudinal healthcare information such as diagnoses, procedures, laboratory test results, and medications. Deep learning algorithms can be effectively used to predict certain medical events by capturing features and patterns contained in EHR data [14]. For example, scalable deep learning algorithms were applied in a previous study to accurately predict mortality and readmission in EHR data from two academic medical centers [15].

In this paper, we investigated the prediction of future CVH from previous measurements of CVH among patients utilizing one type of recurrent neural network (RNN) – long short-term memory (LSTM) networks [16]. LSTM is one type of architecture from recurrent neural network (RNN) which can capture temporal dynamic behavior from a temporal sequence. LSTM architecture is well-suited to predict time series with time lags of unknown size by learning from the previous experiences [17]. Unlike previous studies, we implemented LSTM techniques based on a large and nationally-representative longitudinal dataset of patients. We also compared LSTM models with other two baseline models, i.e., logistic regression (LR) and random forest (RF). The EHR data used for these analyses was from more than 70 outpatient clinics across the US. We included five CVH submetrics (i.e., SMK, BMI, A1C, LDL and BP) in our analyses due to data availability.

Our purpose was to better understand the CVH trajectory of patients and whether steps could be taken by providers and patients to maximize prevention efforts and the worsening of CVH. For example, if the CVH level was predicted to be worsening based upon the previous measures, then providers and patients could better maintain or control CVH to prevent it from becoming worse.

## Methods

The Guideline Advantage (TGA) is an ambulatory quality clinical data registry of EHR data from more than 70 different clinics across the US. The American Cancer Society, the American Diabetes Association, and the AHA established TGA for tracking and monitoring disease management and outpatient preventative care (https://www.scripps.org/sparkle-assets/documents/heart_rhythm_facts.pdf). In this paper, we used TGA data to predict their most recent CVH status based on previous CVH data. Each future CVH submetric was defined as the most recent measurement for each patient, while the previous CVH submetrics comprised all preceding measurements.

We first identified the patients with at least one CVH metric with a result date within a 13-year period (2004–2016) of observation. Among these patients, 230,800 had SMK data, and 53,882 patients had measurements of hemoglobin A1c (A1C) measures, 114,235 cholesterol (low-density lipoprotein, LDL), 163,147 BMI, and 261,526 BP, respectively. We identified 216,445 patients with at least two measures at different dates in any CVH submetric (25,080 patients for A1C, 58,385 patients for LDL, 121,267 patients for BMI, 197,387 patients for BP, and 126,709 patients for SMK).

Each of the five CVH measures as defined above were classified into one of three categories according to Table 1: ideal, intermediate, or poor. We utilized the Multum drug database [19] as a template to convert all drug names to their corresponding drug class. We employed the Levenshtein distance algorithm [20] in the conversion process to compare drug names in our data set with those in the Multum drug database. Medications were considered as treatments for A1C, LDL, and BP only if the Levenshtein distance between compared strings was less than five.

**Table 1:** Measures of CVH which are available in the TGA (Adapted from: Lloyd-Jones, 2011) [18]

	Poor health	Intermediate health	Ideal health
Health behaviors			
Smoking status	Yes	Former ≤12 months	Never or quit > 12 months
Body mass index	≥ 30 kg/m^2^	−5 - 29.9 kg/m^2^	< 25 kg/m^2^
Health factors			
LDL	≥ 160 mg/dL	130–159 mg/dL or treated to goal	< 130 mg/dL
Blood pressure	Systolic ≥140 mmHg or Diastolic ≥90 mmHg	Systolic 120–139 mmHg or Diastolic 80–89 mmHg or treated to goal	Systolic < 120 mmHg Diastolic < 80 mmHg
Fasting plasma glucose	≥ 126 mg/dL	100–125 mg/dL or treated to goal	< 100 mg/dL

**Fig. 1:**
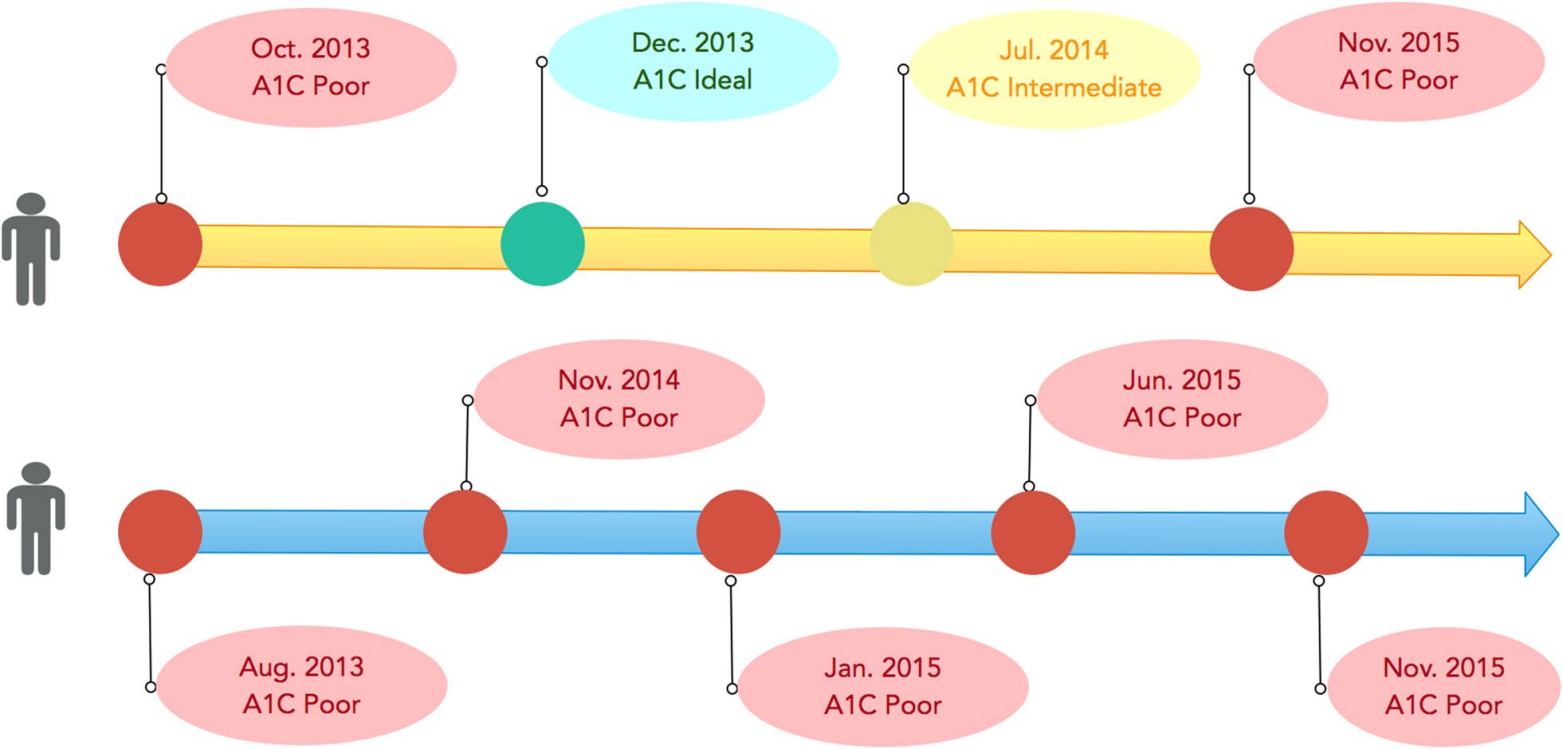
Examples of two random patients with A1C measurements assessed on different dates

All CVH data for each submetric were sorted in a time order as shown in Fig. 1, which illustrates the trajectory of A1C measurements for two random patients. The last CVH category in the series were marked as labels (i.e., ideal, intermediate, or poor A1C). We set each CVH submetric value as 2 for the ideal category, 1 for intermediate category, and 0 for poor category for each CVH submetric label. All of the preceding categorical measures were used as features.

**Fig. 2:**
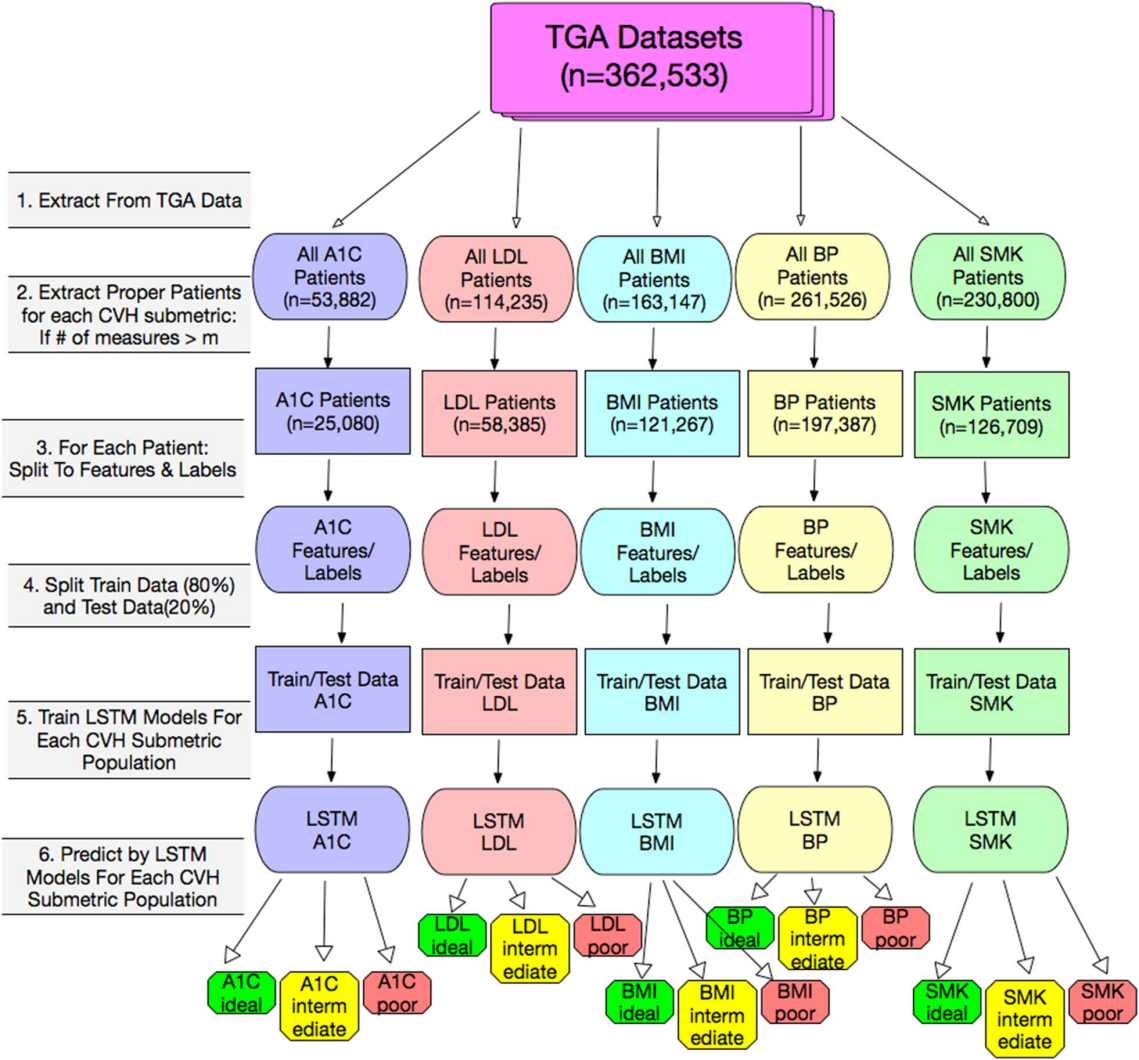
Flowchart of our prediction by LSTM for each CVH submetric from each submetric measures. Here m = 1. Where m is the threshold of number of measures, A1C patients means all the patients who had A1C measures and also number of measures was more than m. The same definition was used for LDL, BMI, BP, and SMK patients

We first conducted predictions for each CVH submetric category by LSTM as shown in Fig. 2.

For each CVH submetric, we selected patients who had at least two measurements and then combined the submetric name with its category. For example, if a patient had a poor category for BMI, we combined the submetric name “bmi” and category “poor” as “bmipoor”. Then we mapped the resulting features to 32-dimensional vectors by a word embedding technique Word2Vec in our model, from which the resulting features (e.g., bmipoor) were all denoted by numerical vectors. The Genism Word2Vec model was configured the hyperparameters as following: size (embedding dimension) as 32, window (the maximum distance between a target word and all words around it) as 5, min_count (the minimum number of words counted when training the model) as 1, sg (the training algorithm) as CBOW (The continues bag of words). We also added time information for all measurements as time steps. Each feature was associated with a time point which was calculated by the difference in days between the corresponding visit time and the latest measurement time. Sex and age information were also added for all patients. The input length was the maximum count of measures among patients in each prediction case. Thus, each patient was represented by a numerical embedding vector. Next, these embedding vectors were fed to a LSTM model. For each submetric, the dataset was randomly split into training dataset (80%) and testing dataset (20%). We used the area under the receiver operator curve (AUC) to evaluate the performance of the LSTM model.

We then applied the same methodology to patients who had at least two measures for each CVH submetric. For this patient subpopulation, we performed two types of predictions: one in which we used only one CVH submetric to predict future values for that category, and the other in which we used all 5 CVH submetrics to predict the 5 future submetrics by multi-label classification and multiclass classification approaches. Criterion of AUC and accuracy were utilized to evaluate the performance of the two types of predictions. Finally, we evaluated the difference in performance for these two types of predictions.

For predictions using all 5 CVH submetric measures, we listed AUC and accuracy for the multilabel classification approach which was designed to predict all 15 classes (5 submetrics × 3 classes each, method 1) and a multiclass classification approach was used to predict all 3 classes of each CVH submetric in which we repeated the analysis 5 times (method 2). We applied another prediction using only one CVH submetric to predict future values for that category. For each subpopulation, we trained our model using 7855 patient samples, and validated the model on 1964 samples. We listed accuracy as the ability to predict correctly for each case as equally important.

To compare the performance of the above methods, we investigated the correlations between the latest status of each of the 5 CVH submetrics. The three levels of each submetric were represented as numerical values (0, 1, 2) corresponding to CVH levels of poor, intermediate, and ideal. All patients were assigned numerical CVH levels for their 5 CVH submetrics. For example, if a patient had ideal A1C, intermediate LDL, poor BMI, poor BP, and ideal SMK, then the levels of the 5 CVH submetrics were represented as 2, 1, 0, 0, and 2. The Pearson’s correlation coefficients were calculated to evaluate correlations between these 5 CVH submetrics.

Our LSTM model was comprised of an input layer, one hidden layer (with 100 dimensions) and an output layer. A categorical cross-entropy loss function was employed as the output layer and a sigmoid function was used as the activation function for the hidden layer. Adam optimizer [21] was used to optimize the model with a mini-batch size of 64 samples. We did an extensive hyperparameter search for activation functions (i.e., Sigmoid, tanh, SeLU and ReLU), as well as the embedding dimensions of 32 or 64. We did not extensively search other hyperparameters such as number of LSTM layers, number of recurrent units, or batch size, as these hyperparameters were of minor importance upon initial investigation [22].

We also compared LSTM models with two baseline models: logistic regression (LR) and random forest (RF). The logistic regression model was configured as follows: the L2 norm was used in the penalization, i.e., the variance of predicted value and real value of training data; the stopping criteria was set as 1.0*10–4; the inverse of regularization strength, which reduces the potential overfitting, was set as 1.0. The RF model was configured as follows: the number of trees in the random forest was set 100; the number of maximum features can be used in each tree was set as the square root of the total number of features; the minimum number of samples at a leaf node of a tree was set as 1. Analyses were conducted by using the libraries of Scikit-learn, Scipy, and Matplotlib with Python, version 3.6.5 in 2019.

## Results

Table 2 shows characteristics of our overall study population and of the subpopulation of patients with multiple measurements for each of the 5 submetrics. The overall population contained approximately 56% females and 48% white race with an average age of 45 years. For the overall population, the average values of CVH submetrics were as follows: A1C was 7.2%, LDL was 107 mg/dL, BMI was 28.5 kg/m^2^, systolic BP was 123 mmHg and diastolic BP was 73 mmHg. Around 16% patients were current smokers. The subpopulation was older (58 years), and BMI and BP were higher compared to the overall population.

**Table 2:** Characteristics [mean (SD) or n (%)] of the overall and common study population

Patients	Overall population (*n* = 216,445)	Subpopulation (*n* = 9819)
*Demographics*		
Gender *n (%)*		
Female	121,592 (56.2)	5423 (55.2)
Male	94,747 (43.8)	4392 (44.7)
Other/Unknown	106 (0.0)	4 (0.1)
Race *n (%)*		
White	103,630 (47.9)	3254 (33.1)
Non-white	46,327 (21.4)	1600 (16.3)
Unknown	67,283 (31.1)	4965 (50.6)
Age, years *mean (std)*	45 (23)	58 (14)
*CVH submetrics mean (std)*		
A1C (%)	7.2 (1.9)	7.4 (1.9)
LDL (mg/dL)	106.9 (35.9)	101.7 (34.8)
BMI (kg/m^2^)	28.5 (9.2)	34.3 (8.6)
BP, systolic (mmHg)	122.5 (19.4)	129.5 (18.5)
BP, diastolic (mmHG)	73.0 (16.1)	76.8 (11.7)
Current smoking *n (%)*	34,122 (15.8)	2667 (27.2)

**Fig. 3:**
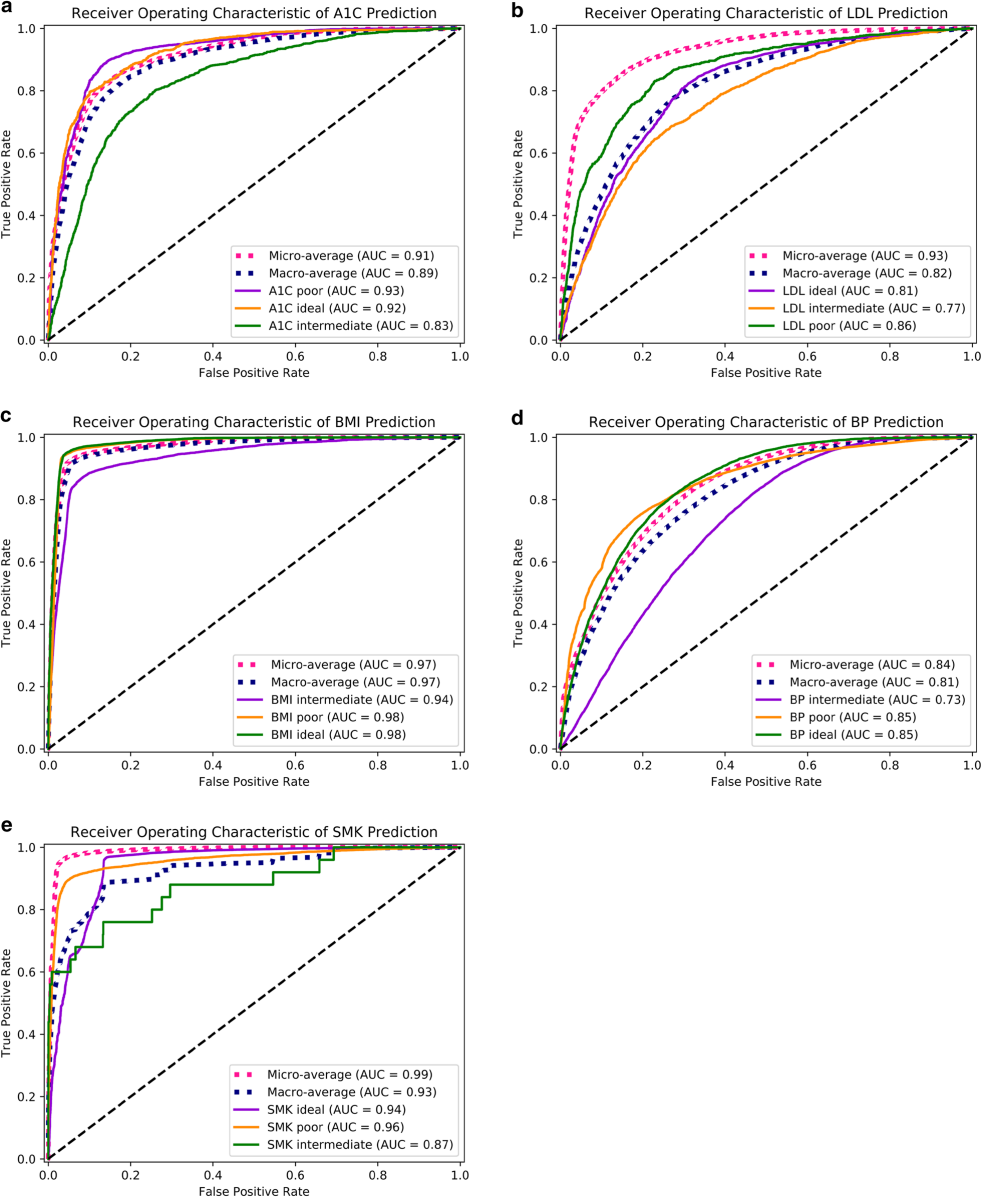
The area under the curve (AUC) for predictions regarding 5 CVH submetrics by LSTM. Figure (**a**) shows the prediction for A1C according to all previous A1C measures, and **b-e** were for LDL, BMI, BP, and SMK predictions, respectively

Figure 3 displays the performance of the LSTM models for each CVH submetric prediction. The AUC for the A1C category prediction using all measures was 0.21 for ideal category, 0.83 for intermediate category, and 0.93 for poor category; the micro-average and macro-average of AUC for A1C prediction was 0.91 and 0.89, respectively (Fig. 3a). Similarly, the micro-average and macro-average AUC for LDL prediction was 0.93 and 0.82 (0.97 and 0.97 for BMI, 0.84 and 0.81 for BP, 0.99 and 0.93 for SMK). The values of AUC for LDL predictions for ideal, intermediate, and poor categories were 0.81, 0.77 and 0.86 (0.98, 0.94 and 0.98 for BMI, 0.85, 0.73 and 0.85 for BP, 0.94, 0.87 and 0.96 for SMK), respectively (Fig. 3b–e). Additional file 1: Figure S1 and Additional file 2: S2 (in the supplementary material section) displays the performance of LR and RF by ROC curves for each submetric.

Table 3 lists other additional metrics, i.e., overall accuracy, precision, recall, and F1-score, to evaluate performance for LSTM, LR, and RF models. The results showed that LR performed better than RF in all cases, and LSTM performed the best in all the cases.

**Table 3:** Additional metrics used to evaluate each model

Models	Submetric cases	Accuracy	Precision	Recall	F1-score
LSTM	A1C ideal	0.78	0.74	0.68	0.71
	A1C intermediate		0.66	0.66	0.66
	A1C poor		0.87	0.89	0.88
	LDL ideal	0.80	0.83	0.97	0.89
	LDL intermediate		0.46	0.12	0.19
	LDL poor		0.49	0.32	0.38
	BMI ideal	0.91	0.94	0.95	0.94
	BMI intermediate		0.83	0.83	0.83
	BMI poor		0.94	0.94	0.94
	BP ideal	0.65	0.59	0.24	0.34
	BP intermediate		0.53	0.70	0.61
	BP poor		0.78	0.75	0.77
	SMK ideal	0.95	0.97	0.97	0.97
	SMK intermediate		0.86	0.24	0.38
	SMK poor		0.87	0.85	0.86
RF	A1C ideal	0.78	0.77	0.61	0.68
	A1C intermediate		0.64	0.70	0.67
	A1C poor		0.87	0.89	0.88
	LDL ideal	0.79	0.81	0.98	0.89
	LDL intermediate		0.0	0.0	0.0
	LDL poor		0.46	0.35	0.40
	BMI ideal	0.91	0.92	0.94	0.93
	BMI intermediate		0.82	0.81	0.81
	BMI poor		0.95	0.93	0.94
	BP ideal	0.63	0.66	0.06	0.11
	BP intermediate		0.52	0.69	0.59
	BP poor		0.74	0.78	0.76
	SMK ideal	0.93	0.97	0.95	0.96
	SMK intermediate		0.0	0.0	0.0
	SMK poor		0.80	0.85	0.82
LR	A1C ideal	0.78	0.74	0.68	0.71
	A1C intermediate		0.66	0.66	0.66
	A1C poor		0.87	0.89	0.88
	LDL ideal	0.79	0.83	0.96	0.89
	LDL intermediate		0.45	0.16	0.24
	LDL poor		0.47	0.27	0.34
	BMI ideal	0.91	0.94	0.94	0.94
	BMI intermediate		0.83	0.83	0.83
	BMI poor		0.94	0.94	0.94
	BP ideal	0.65	0.55	0.26	0.36
	BP intermediate		0.54	0.62	0.58
	BP poor		0.75	0.79	0.77
	SMK ideal	0.95	0.96	0.97	0.97
	SMK intermediate		0.80	0.16	0.27
	SMK poor		0.86	0.83	0.85

Table 4 lists the AUC and accuracy for the prediction of future CVH by LSTM models by using all five submetric measures and single CVH submetrics as predictors for the subpopulation.

**Table 4:** AUC and accuracy by LSTM using all 5 CVH submetric measures using multi-label (Method 1), multiclass (Method 2), and single submetric measures for the subpopulation

CVH	AUC			Accuracy (%)		
CVH category	Method 1	Method 2	Single	Method 1	Method 2	Single
A1C ideal	0.97	0.92	0.91	99.7	90.7	90.3
A1C intermediate	0.81	0.83	0.84	94.7	78.7	79.7
A1C poor	0.90	0.92	0.93	50.7	86.2	87.4
LDL ideal	0.77	0.78	0.78	60.7	84.0	82.3
LDL intermediate	0.69	0.71	0.73	99.9	88.0	88.4
LDL poor	0.82	0.86	0.81	1.0	92.2	93.1
BMI ideal	0.97	0.97	0.97	97.6	96.6	97.0
BMI intermediate	0.96	0.95	0.94	97.6	92.0	91.8
BMI poor	0.98	0.97	0.97	69.3	94.6	94.3
BP ideal	0.76	0.77	0.77	99.5	75.9	76.1
BP intermediate	0.59	0.61	0.63	99.0	59.3	58.8
BP poor	0.78	0.77	0.79	99.9	81.2	80.2
SMK ideal	0.95	0.94	0.93	45.4	94.7	94.3
SMK intermediate	1.0	0.78	1.0	1.0	99.7	99.9
SMK poor	0.95	0.95	0.93	98.3	94.7	94.3

Table 4 showed that AUC and accuracy values remained almost the same as predictions using all 5 CVH submetrics compared to only using data from a single CVH submetric. There were some differences between methodologies. For accuracy values for multi-label classification (Method 1), which predicted all 15 classes at one time, the model calculated an overall optimal score while ignoring some classes. As a result, there were some low values of accuracy which resulted from this method; for example, accuracy of predicting poor A1C was 50.7%.

**Fig. 4:**
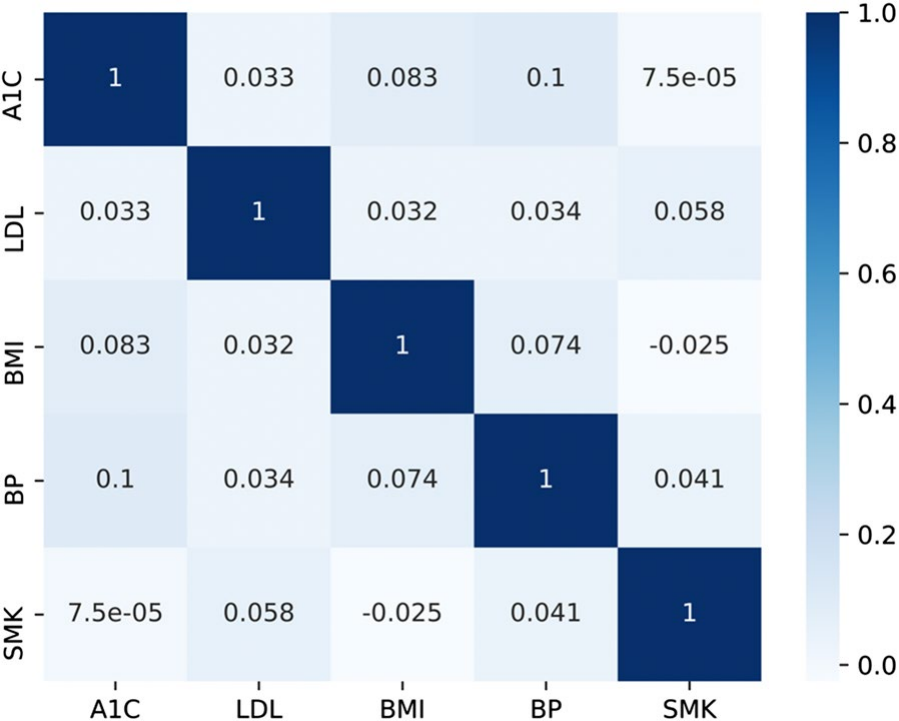
Correlations between 5 CVH submetric labels

Figure 4 lists the correlations between the latest status of each of the 5 CVH submetrics. The calculated values were Pearson’s correlation coefficients. Higher absolute values indicated stronger associations between variables. The correlation coefficient between A1C and BP was 0.1, 0.083 for A1C and BMI, and 0.074 for BMI and BP.

## Discussion

In this study, we employed a RNN model – an LSTM model – to predict CVH measure categories in each submetric using 14-year longitudinal CVH measures from the EHR of more than 70 different outpatient clinics. We also studied a subpopulation of patients who had at least two measures for each CVH submetric to predict the CVH submetric measure categories using data from a single submetric data versus all 5 submetrics employing multi-label and multiclass classification techniques.

The comparison of LSTM models to two baseline models (i.e., LR and RF) indicated that LSTM models outperformed the other models. Our results indicated that CVH measure categories can be accurately predicted by previous CVH measures for that metric with LSTM models. BMI, SMK, A1C, and LDL predictions and all their corresponding micro-average AUC values were greater than 0.90. The micro-average AUC value for BP prediction was lower (0.84), which might result from the measurement variability of BP inhereherin EHR data.

We used all previous trajectories of a given CVH submetric to predict the most recent value for that submetric. We also used all available CVH submetrics to predict the most recent value for a given submetric considering there might be associations between different submetrics. Our results indicated that combining all CVH submetrics did not improve the prediction performance. Thus, the focus of our study was to predict one CVH submetric according to trajectories of the same submetric. In our subpopulation analyses, the criterion of AUC and accuracy were not improved by using all 5 CVH submetrics compared to data from a single submetric. One reason for this finding may be due to the low correlations between these 5 submetric labels.

One key strength of our study was the use of the TGA dataset which was a large and nationally representative longitudinal EHR dataset. The contribution of this study was the first to investigate the predictions of future CVH from all previous CVH measures by deep learning approaches using TGA longitudinal EHR data. We acknowledge that the conversion of numerical variables into categories may result in information loss, therefore our future work would focus on using the numerical values to predict the three classes.

LSTM models can effectively predict CVH trajectories of patients on a large ambulatory population. It is crucial to predict future changes in CVH to better manage CVH of patients. For example, if the A1C level was predicted to be worsening based upon the previous measures, then providers and patients could better maintain or control A1C to prevent it from becoming worse. Therefore, predicting future CVH could improve the health and quality of life of patients. Ideal CVH measures were associated with lower incidence of CVD and cancers [6, 23–28]. Thus, predicting future levels of CVH might indirectly decrease the cost and burden on the health system caused by CVD and cancers.

## Limitations

A limitation in our analyses was that our results were based on the TGA data source, which is large and more representative, thus it might yield to different results on other data source.

## Conclusions

We found that LSTM models can be effective at accurately predicting CVH measure categories in each submetric from the time-series CVH measures. The performance was not improved by using all 5 CVH submetric measures compare to using single submetric measures in the subpopulation. Predicting patients’ future CVH levels might increase patient CVH health and indirectly improve quality of life for patients and decrease the burden and cost for clinical health system caused by CVD and cancers. These findings have important implications for predicting trajectories of CVH in a patient population. Future research should work towards identifying optimal time to intervene on future CVH values.

## Supplementary Information

**Additional file 1: Figure S1**. The area under the curve (AUC) for predictions regarding 5 CVH submetrics by LR models. Figure (**A**) shows the prediction for A1C according to all the previous A1C measures, and (**B**)-(**E**) were for LDL, BMI, BP, and SMK predictions, respectively.

**Additional file 2: Figure S2**. The area under the curve (AUC) for predictions regarding 5 CVH submetrics by RF models. Figure (**A**) shows the prediction for A1C according to all the previous A1C measures, and (**B**)-(**E**) were for LDL, BMI, BP, and SMK predictions, respectively.

## Data Availability

The datasets belong to a third-party and the authors do not have permission to share the data. Researchers need to apply from American heart association for the access to the datasets.

## Abbreviations

CVD: Cardiovascular disease
CVH: Cardiovascular health
EHR: Electronic health records
TGA: The Guideline Advantage
SMK: Smoking status
BMI: Body mass index
BP: Blood pressure
A1C: Hemoglobin A1c
LDL: Low-density lipoprotein
LSTM: Long short-term memory
AUROC: Area under the receiver operator curve
AHA: American heart association
RNN: Recurrent neural networks
LR: Logistic regression
RF: Random forest
AUC: Area under curve
