# Supplementary material for: Predicting cardiovascular health trajectories in time-series electronic health records with LSTM models
Source: BMC Med Inform Decis Mak. 2021 Jan 6;21:5. doi: 10.1186/s12911-020-01345-1 (PMC7789405; doi:10.1186/s12911-020-01345-1)
Supplement: Supplementary file 2 — Additional file 2: Figure S2. The area under the curve (AUC) for predictions regarding 5 CVH submetrics by RF models. Figure (A) shows the prediction for A1C according to all the previous A1C measures, and (B)-(E) were for LDL, BMI, BP, and SMK predictions, respectively. [file 12911_2020_1345_MOESM2_ESM.pdf]

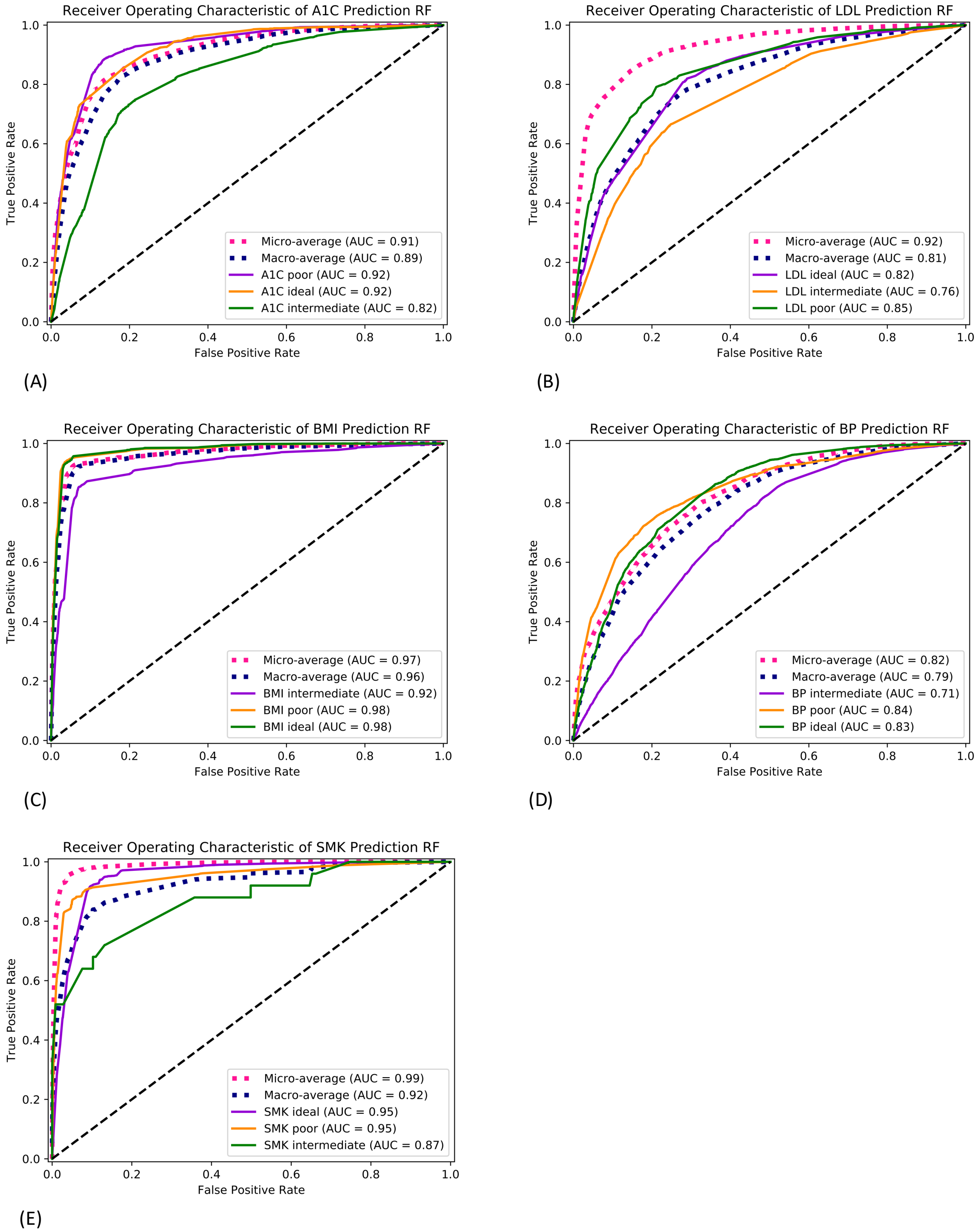

Figure S2. The area under the curve (AUC) for predictions regarding 5 CVH submetrics by RF models. Figure **(A)** shows the prediction for A1C according to all the previous A1C measures, and **(B)-(E)** were for LDL, BMI, BP, and SMK predictions, respectively.
